# Attitudes towards chiropractic: a repeated cross-sectional survey of Canadian family physicians

**DOI:** 10.1186/s12875-021-01535-4

**Published:** 2021-09-15

**Authors:** Jason W. Busse, Sushmitha Pallapothu, Brian Vinh, Vivienne Lee, Lina Abril, Albana Canga, John J. Riva, Daniel Viggiani, Marc Dilauro, Marie-Pierre Harvey, Isabelle Pagé, Avneet K. Bhela, Serena Sandhu, Oluwatoni Makanjuola, Muhammad Taaha Hassan, Ainsley Moore, Claude A. Gauthier, David J. Price

**Affiliations:** 1 Department of Health Research Methods, Evidence, and Impact, McMaster University, Hamilton, ON Canada; 2 Department of Anesthesia, Michael G. DeGroote School of Medicine, McMaster University, HSC-2V9, 1280 Main St. West, Hamilton, ON L8S 4K1 Canada; 3 Department of Biology, McMaster University, Hamilton, ON Canada; 4 Department of Health Sciences, McMaster University, Hamilton, ON Canada; 5 The School of Interdisciplinary Science, McMaster University, Hamilton, ON Canada; 6 Institute for Work & Health, Toronto, ON Canada; 7 Department of Family Medicine, McMaster University, Hamilton, ON Canada; 8 Department of Kinesiology, University of Waterloo, Waterloo, ON Canada; 9 Michael G. DeGroote School of Medicine, McMaster University, Hamilton, ON Canada; 10 Département de chiropratique, Université du Québec à Trois-Rivières, QC Trois-Rivières, Canada; 11 Center for Interdisciplinary Research in Rehabilitation and Social Integration (Cirris), Quebec City, QC Canada; 12 Department of Life Sciences, McMaster University, Hamilton, ON Canada; 13 Claude A. Gauthier, Private Practice, Gatineau, QC Canada

**Keywords:** General practice, Chiropractic, Surveys and questionnaires, Interprofessional relations, Physicians, Attitude of health personnel, Survey

## Abstract

**Background:**

Many primary care patients receive both medical and chiropractic care; however, interprofessional relations between physicians and chiropractors are often suboptimal which may adversely affect care of shared patients. We surveyed Canadian family physicians in 2010 to explore their attitudes towards chiropractic and re-administered the same survey a decade later to explore for changes in attitudes.

**Methods:**

A 50-item survey administered to a random sample of Canadian family physicians in 2010, and again in 2019, that inquired about demographic variables, knowledge and use of chiropractic. Imbedded in our survey was a 20-item chiropractic attitude questionnaire (CAQ); scores could range from 0 to 80 with higher scores indicating more positive attitudes toward chiropractic. We constructed a multivariable regression model to explore factors associated with CAQ scores.

**Results:**

Among eligible physicians, 251 of 685 in 2010 (37% response rate) and 162 of 2429 in 2019 (7% response rate) provided a completed survey. Approximately half of respondents (48%) endorsed a positive impression of chiropractic, 27% were uncertain, and 25% held negative views. Most respondents (72%) referred at least some patients for chiropractic care, mainly due to patient request or lack of response to medical care. Most physicians believed that chiropractors provide effective therapy for some musculoskeletal complaints (84%) and disagreed that chiropractic care was beneficial for non-musculoskeletal conditions (77%). The majority agreed that chiropractic care was a useful supplement to conventional care (65%) but most respondents (59%) also indicated that practice diversity among chiropractors presented a barrier to interprofessional collaboration.

In our adjusted regression model, attitudes towards chiropractic showed trivial improvement from 2010 to 2019 (0.31 points on the 80-point CAQ; 95%CI 0.001 to 0.62). More negative attitudes were associated with older age (− 1.55 points for each 10-year increment from age 28; 95%CI − 2.67 to − 0.44), belief that adverse events are common with chiropractic care (− 1.41 points; 95% CI − 2.59 to − 0.23) and reported use of the research literature (− 6.04 points; 95% CI − 8.47 to − 3.61) or medical school (− 5.03 points; 95% CI  − 7.89 to − 2.18) as sources of knowledge on chiropractic. More positive attitudes were associated with endorsing a relationship with a specific chiropractor (5.24 points; 95% CI 2.85 to 7.64), family and friends (4.06 points; 95% CI 1.53 to 6.60), or personal treatment experience (4.63 points; 95% CI 2.14 to 7.11) as sources of information regarding chiropractic.

**Conclusions:**

Although generally positive, Canadian family physicians’ attitudes towards chiropractic are diverse, and most physicians felt that practice diversity among chiropractors was a barrier to interprofessional collaboration.

**Supplementary Information:**

The online version contains supplementary material available at 10.1186/s12875-021-01535-4.

## Background

One in eight Canadians report attending a chiropractor in the past year, primarily for low back or neck pain [1, 2], and many patients receive care from both a family physician and a chiropractor during the course of their complaint [3]. Medicine and chiropractic, however, have a contentious history [4]. In 1972, the Canadian Medical Association reaffirmed its policy that physicians may not make referrals to chiropractors or acquire x-rays on behalf of chiropractors [5], and until 1983 the American Medical Association held that it was unethical for medical doctors to associate with chiropractors [6]. Some evidence suggests that integrated models of care, in which physicians and chiropractors work in the same clinic, enhance care coordination, referral between disciplines, and trust among providers [7]. Although family physicians have become more accepting of chiropractic [8, 9], current interprofessional relationships between family physicians and chiropractors remain suboptimal [10–14].

Chiropractic in Canada exists on a spectrum. While most providers focus on management of musculoskeletal complaints, approximately 1 in 5 Canadian chiropractors adhere to vitalist traditions of chiropractic which maintain that malpositioned spinal vertebrae (subluxations) interfere with the nervous system causing disease [15]. Vitalist practitioners in Canada are more likely to hold anti-vaccination beliefs, less likely to adhere to guideline recommendations for use of radiographic imaging [15], and receive fewer referrals from physicians [16]. This schism within the profession has been longstanding [17], and some opinion leaders have argued for formally separating the chiropractic profession into evidence-based and vitalist factions [18].

Many patients do not reveal their use of chiropractic to their primary care physician, in part over concerns of disapproval [19]. When patients do report receipt of chiropractic care, communication between physicians and chiropractors is often poor [20]. Understanding how family physicians view chiropractic may provide opportunities to enhance interprofessional relations and improve care of shared patients. The aim of the current study was to survey the attitudes of Canadian family physicians towards chiropractic in 2010 and re-administer the same survey a decade later to explore for changes in attitudes. We hypothesized that family physicians’ attitudes towards chiropractic would show improvement between survey administrations.

## Methods

### Questionnaire development

With the assistance of epidemiologists and content experts, and reference to the previous literature [8, 10, 21–23], we developed a 50-item, English and French-language questionnaire to examine family physicians’ attitudes towards chiropractic (Additional file 1). The final questionnaire provided response options as checkboxes, as a previous report has shown that closed-ended questions result in fewer incomplete questionnaires than open-ended formats [24].

We pre-tested our survey with three family physicians, two clinicians with both medical and chiropractic training, and two chiropractors, to evaluate if the questionnaire adequately measured attitudes towards chiropractic, and if the individual questions adequately reflected the domains of formation of attitudes, referral practices, and impressions towards chiropractic. The pre-test participants also commented on the clarity and comprehensiveness of the questionnaire and the time required for completion.

Thirty survey questions requested demographic data from respondents and asked about their knowledge of chiropractic and referral practices for chiropractic care. The survey also included a 20-item chiropractic attitude questionnaire (CAQ). Each of the 20 questions comprising the CAQ was graded on a 5-point Likert scale (i.e., strongly agree, agree, undecided, disagree, strongly disagree), from 0 to 4. After re-coding so that all reply options were qualitatively in the same direction, the responses were summed to arrive at a total score ranging from 0 (most negative attitude towards chiropractic) to 80 (most positive attitude towards chiropractic). The internal consistency of the CAQ, using all respondents from both administrations, was high (Cronbach’s alpha, 0.83). The last item of the CAQ asked about the respondent’s general attitude towards chiropractic and served as an embedded validation question. The Spearman correlation between responses to this item and the total CAQ score (excluding the last question) was 0.85 (*p* < 0.01), further supporting construct validity of the CAQ.

### Questionnaire administration

We used the 2009 Scott’s Canadian Medical Directory [25] to acquire a random sample of 1000 Canadian family physicians with a random-number generator. The Scott’s Directory contains telephone and fax numbers for physicians, but email addresses are infrequently provided. Between October and December 2010, all physicians’ offices were called to establish if they were in active practice, confirm a working fax number, and inquire if an English or French-language survey was preferred. Eligible physicians (those in active practice and for whom a working fax number was identified) were sent a survey by fax. Recipients were provided with a disclosure letter detailing the intent of the survey and explicit instructions that, should they choose not to complete the survey, they could provide this decision by fax or email to avoid further requests. Therefore, informed consent was implied if physicians provided a completed survey.

At 4 and 8 weeks following the initial survey, we re-faxed the questionnaire to all non-responders (i.e., those fax numbers from which we did not receive a completed survey) unless they indicated they did not wish to participate. We telephoned each office that received a 3rd (final) survey prior to faxing to encourage completion of the instrument, which has been shown to improve response rates [26]. Our survey was performed in accordance with the Declaration of Helsinki and approved by the Hamilton Integrated Research Ethics Board (project no. 10–305), and all methods were performed in accordance with relevant guidelines and regulations [27].

We subsequently used the 2019 Scott’s Canadian Medical Directory [22] to acquire a random sample of 2996 Canadian family physicians selected using a computer-based random number generator. From September to November 2019, we administered the same 50-item survey to physicians in this sample who were in active practice and for whom we confirmed a working fax number, in the same manner as in 2010. The Hamilton Integrated Research Ethics Board granted approval for re-administration of our survey (project no. 7355).

### Data management and storage

Members of our study team transferred information from surveys with single-key entry, as they were received, to an electronic database (SPSS) on a password-protected computer. Data was checked by a second team member for inconsistencies or unusual answers (e.g., age > 100). Once data was entered and verified, all paper surveys were shredded and disposed of.

### Statistical analysis

We generated frequencies for all collected data and, for purposes of presentation, collapsed responses to individual CAQ items into agree (strongly agree + agree), undecided, and disagree (strongly disagree + disagree). We reported categorical data as proportions and continuous data as means and standard deviations (SDs) if normally distributed and as medians and interquartile ranges (IQRs) if not. To reduce the risk of spurious associations due to multiple testing, we identified any individual question within the CAQ in which the proportion of respondents who agreed or disagreed changed by ≥10% between the 2010 and 2019 survey administrations and used an independent samples Mann-Whitney U test to explore for statistical significance.

Based on previous surveys [28–30], we hypothesized, a priori, the following associations of respondents’ attitudes towards chiropractic: (1) older physicians would hold more negative attitudes; (2) more positive attitudes if they saw a greater proportion of patients with musculoskeletal complaints; (3) physician’s endorsing patient feedback, a relationship with a specific chiropractor, personal treatment experience, or feedback from family and friends as sources of information on chiropractic would hold more positive attitudes; and (4) physician’s endorsing the scientific literature, professors, the media, or lectures during medical school as sources of information on chiropractic would hold more negative attitudes. We also hypothesized that re-administration of the survey in 2019 would show more positive attitudes versus the original administration in 2010. These variables were entered into a generalized linear model. The dependent variable, attitude towards chiropractic, was defined as the aggregate score of the CAQ. We calculated that we would require at least 110 completed surveys to ensure that our regression model was reliable (10 respondents for each independent variable considered) [31].

All comparisons were 2-tailed and an independent factor was considered statistically significant if it had a *p*-value < 0.05 in the final multivariable model. We report the unstandardized regression coefficient and 95% confidence interval (CI) for each variable in our regression model. The value of the unstandardized regression coefficient represents the change in response score on the 80-point CAQ. Multicollinearity was deemed concerning if the variance inflation factor for any independent variable was greater than five [32]. We performed all analyses using IBM SPSS 26.0 statistical software (Armonk, NY: IBM Corp).

## Results

### Characteristics of respondents

In 2010, among 685 of 1000 family physicians who were in active practice and for whom we confirmed a working fax number and sent our survey, 251 returned a completed questionnaire (37% response rate; Additional file 1: Fig. 1). Among 2429 eligible family physicians identified in 2019, 162 provided a completed survey for a 7% response rate (Additional file 1: Fig. 2).

The mean age of respondents was 50 (SD 10) and 56% were men, although there was a higher prevalence of women in the more recent survey (40% in 2010 and 49% in 2019). Most respondents had been active clinically for > 20 years and worked in a community-based practice focused on general family medicine. Most physicians attended to patient populations of which > 30% presented with musculoskeletal complaints (Table 1).

**Table 1:** Demographic characteristics of respondents

Year of administration	2010	2019
No. of respondents	251	162
Age, mean (SD)	51 (10)	50 (10)
Gender, n (%) ^a^		
Male	150 (60%)	80 (51%)
Female	101 (40%)	77 (49%)
Years in practice, n (%)		
< 5 years	19 (8%)	14 (9%)
5 to 10 years	34 (14%)	25 (15%)
11 to 20 years	50 (20%)	36 (22%)
> 20 years	148 (59%)	87 (54%)
Country of origin, n (%) ^b^		
Canada	193 (78%)	104 (67%)
United States	6 (2%)	2 (1%)
Other	49 (20%)	50 (32%)
Practice environment, n (%) ^c^		
Community	153 (61%)	116 (72%)
Private practice	130 (52%)	55 (34%)
Hospital-based	55 (22%)	47 (29%)
Multidisciplinary	45 (18%)	32 (20%)
Academic	31 (12%)	16 (10%)
Patient population with musculoskeletal complaints, n (%) ^d^		
< 10%	5 (2%)	26 (16%)
10 to 20%	46 (19%)	41 (25%)
21 to 30%	71 (29%)	34 (21%)
31 to 40%	59 (24%)	29 (18%)
41 to 70%	58 (23%)	52 (32%)
> 70%	10 (4%)	7 (3%)
Clinical area, n (%) ^d^		
General family	236 (94%)	145 (90%)
Geriatrics	52 (21%)	29 (18%)
Pediatrics	48 (19%)	19 (12%)
Palliative care	45 (18%)	32 (20%)
Emergency medicine	44 (18%)	39 (24%)
Obstetrics & gynecology	39 (16%)	22 (14%)
Psychotherapy	38 (15%)	13 (8%)
Pain medicine	34 (14%)	24 (15%)
Sports medicine	33 (13%)	16 (10%)
Occupational medicine	14 (6%)	5 (3%)
Anesthesia	7 (3%)	4 (3%)

^a^ total number of respondents was 157 for the 2019 survey

^b^ total number of respondents was 156 for the 2019 survey

^c^ total number of respondents was 249 for the 2010 survey

^d^ total percentage is > 100% as respondents could choose more than one option

### Knowledge of and experience with chiropractic

Respondents endorsed multiple sources of information regarding chiropractic, but feedback from their patients was the most common. Seventy-one percent of family physicians reported referring patients for chiropractic care and, among these, most referred ≤25 patients per year. Referrals were usually prompted by patient request (57%; 237 of 413) or non-response to medical treatment (40%; 166 of 413) (Table 2).

**Table 2:** Family physician’s sources of information on chiropractic and referral practices

Year of administration	2010	2019
No. of respondents	251	162
Sources of information on chiropractic, n (%)^a^		
Patient feedback	210 (84%)	121 (75%)
Relationship with a specific chiropractor	105 (42%)	51 (32%)
Research literature	94 (38%)	67 (41%)
Personal treatment experience	85 (34%)	66 (41%)
Family and friends	79 (32%)	48 (30%)
Medical school	50 (20%)	39 (24%)
Media	44 (18%)	21 (13%)
Professors/supervisors/mentors	43 (17%)	29 (18%)
Residency	11 (4%)	10 (6%)
Frequency of patient referral for chiropractic treatment, n (%) ^b^		
Daily	3 (1%)	1 (1%)
Weekly	46 (18%)	26 (17%)
Monthly	79 (32%)	51 (33%)
Every year	56 (22%)	30 (19%)
Never	67 (27%)	49 (31%)
Number of patients referred for chiropractic care per year, n (%) ^c^		
1 to 10	86 (34%)	57 (36%)
11 to 25	52 (21%)	34 (21%)
26 to 50	32 (13%)	17 (11%)
> 50	14 (6%)	10 (6%)
None	67 (27%)	42 (26%)
Reason for chiropractic referral, n (%) ^a,d^		
Patient request	140 (56%)	97 (68%)
Non-response to medical treatment	103 (41%)	63 (44%)
Literature supports chiropractic care	73 (29%)	41 (29%)
Relationship with a specific chiropractor	57 (23%)	29 (20%)
Personal experience as a chiropractic patient	29 (12%)	23 (16%)
Other reasons	17 (7%)	9 (6%)

^a^ total percentage is > 100% as respondents could choose more than one option

^b^ total number of respondents was 157 for the 2019 survey

^c^ total number of respondents was 169 for the 2019 survey

^d^ respondents are limited to the family physicians that reported referring patients for chiropractic

Only 13% of physicians (53 of 413) worked in a multidisciplinary environment where chiropractic care was available, and 40% (165 of 413) had sought chiropractic care for themselves. Most had not received information on chiropractic during their medical training, and the majority (80%) felt their education should (52%; 214 of 413) or possibly should (28%; 115 of 413) include such information. Most respondents’ opinions on chiropractic were formed after medical school (82%; 337 of 413), and most (51%; 209 of 413) described themselves as a little knowledgeable. In 2010, most respondents (52%) felt that adverse events were uncommon with chiropractic care, and in 2019 most physicians believed that adverse events were common but serious events were rare (47%). In 2010, most respondents (46%) were very comfortable discussing chiropractic with their patients, whereas in 2019 most (41%) were only somewhat comfortable (Additional file 1: Table 1).

Fifteen percent (62 of 413) of physicians felt that chiropractic care should be available in multidisciplinary settings (29% were unsure), and 25% felt that chiropractic should be available in hospitals, either with (17%; 69 of 413) or without (8%; 34 of 413) physician referral. Respondents varied on whether chiropractic care should be offset by government funding: 35% agreed, 33% were unsure, and 27% disagreed. Forty-three percent of family physicians definitely (17%) or somewhat (26%) perceived chiropractors as primary care providers, and most (81%; 335 of 413) wanted consultation notes from chiropractors who attended their patients. Seventy-five percent of respondents had received requests from chiropractors to refer patients for imaging studies. Most physicians (59%; 245 of 413) believed that practice diversity within the chiropractic profession was a barrier to interprofessional collaboration. (Additional file 1: Table 2).

### Attitudes towards chiropractic

Forty-eight percent of family physicians (198 of 413) endorsed a positive impression of chiropractic, 27% were unsure, and 25% held negative views. Respondents endorsing a positive view had an average CAQ score of 50.2 out of 80 (SD 7.5), undecided respondents had an average CAQ score of 39.4 (SD 5.8), and physicians with negative impressions had a mean CAQ score of 24.9 (SD 9.1). An important change in continuous outcome measures can be estimated as half a SD of the aggregate score for a given population [33], and by this standard, a 6-point difference on the CAQ would be considered meaningful.

Responses to individual items on the CAQ are provided in Table 3. Most physicians felt that chiropractors provide effective management for some musculoskeletal disorders (84%), that chiropractic was a useful supplement to medical care (65%), and chiropractors could reduce patient overload for family physicians (52%). Many physicians endorsed that chiropractors provide a patient-centred approach (45%) and use approaches from which medicine could benefit (43%). Alternately, most respondents disagreed that chiropractic was effective for non-musculoskeletal conditions (77%) and were unsure whether chiropractors treat in accordance with evidence-based practices (52%). Many felt that chiropractic manipulation of the neck was unsafe (47%) and 37% agreed that chiropractors provide patients with misinformation regarding vaccination.

**Table 3:** Responses to the chiropractic attitude questionnaire (*n* = 251 in 2010; *n* = 162 in 2019)

Item	Agree, n (%)		Undecided, n (%)		Disagree, n (%)	
	2010	2019	2010	2019	2010	2019
Chiropractors promote unnecessary treatment plans	121 (48%)	65 (40%)	86 (34%)	52 (32%)	44 (18%)	45 (28%)
Chiropractors provide effective therapy for some musculoskeletal conditions	216 (86%)	130 (80%)	20 (8%)	20 (12%)	15 (6%)	12 (7%)
Chiropractors make excessive use of radiographic imaging	83 (33%)	58 (36%)	107 (43%)	57 (35%)	61 (24%)	47 (29%)
Chiropractors provide a patient centered approach	112 (45%)	75 (46%)	111 (44%)	70 (43%)	28 (11%)	17 (11%)
I have to spend time correcting erroneous information patients have received from chiropractors	81 (32%)	53 (33%)	48 (19%)	33 (20%)	122 (49%)	76 (47%)
Chiropractic manipulation of the neck is generally a safe therapy	59 (24%)	37 (23%)	70 (28%)	54 (33%)	122 (49%)	71 (44%)
Chiropractors can provide effective therapy for some non- musculoskeletal conditions (e.g. asthma, colic, etc.)	13 (5%)	9 (6%)	37 (15%)	37 (23%)	201 (80%)	116 (72%)
Family physicians may risk professional liability if they refer a patient to a chiropractor	50 (20%)	36 (22%)	74 (30%)	54 (33%)	127 (51%)	72 (44%)
Chiropractors can reduce patient overload for family physicians with respect to patients with musculoskeletal complaints	119 (47%)	94 (58%)	62 (25%)	34 (21%)	70 (28%)	34 (21%)
Chiropractors provide patients with misinformation regarding vaccination	96 (38%)	58 (36%)	120 (48%)	74 (46%)	35 (14%)	30 (19%)
Chiropractic provides effective therapy for post-surgical rehabilitation	35 (14%)	38 (24%)	152 (61%)	81 (50%)	64 (26%)	43 (27%)
Chiropractors lack sufficient clinical training	44 (18%)	25 (15%)	110 (44%)	60 (37%)	97 (39%)	77 (48%)
Chiropractic care is a useful supplement to conventional medicine	163 (65%)	106 (65%)	55 (22%)	30 (19%)	33 (13%)	26 (16%)
Chiropractors engage in overly aggressive marketing	107 (43%)	57 (35%)	95 (38%)	51 (31%)	49 (20%)	54 (33%)
Chiropractic includes ideas and methods from which conventional medicine could benefit	109 (43%)	68 (42%)	84 (34%)	62 (38%)	58 (23%)	32 (20%)
The results of chiropractic manipulation are due to the placebo effect	33 (13%)	23 (14%)	89 (36%)	62 (38%)	129 (51%)	77 (48%)
Chiropractors treat in accordance with evidence-based practices	36 (14%)	42 (26%)	141 (56%)	75 (46%)	74 (30%)	45 (28%)
Chiropractic has no role in the routine care of my patients	75 (30%)	36 (22%)	43 (17%)	35 (22%)	133 (53%)	91 (56%)
Chiropractic breeds dependency in patients on short-term symptomatic relief	88 (35%)	55 (34%)	68 (27%)	52 (32%)	95 (38%)	55 (34%)
Overall, my impression of chiropractic is favorable	118 (47%)	80 (49%)	68 (27%)	43 (27%)	65 (26%)	39 (24%)

There were 5 items on the CAQ in which the proportion of respondents who agreed or disagreed shifted by ≥10% between administrations in 2010 and 2019, of which three were statistically significant. Canadian family physicians surveyed in 2019 were more likely to: (1) disagree that chiropractors promote unnecessary treatment plans (28% in 2019 vs. 18% in 2010; *p* < 0.001), (2) agree that chiropractors provide effective care for post-surgical rehabilitation (24% vs. 14%; *p* = 0.05), and (3) agree that chiropractors treat in accordance with evidence-based practices (26% vs. 14%; p = 0.05).

In our adjusted regression model, overall impressions towards chiropractic showed trivial improvement from 2010 to 2019 (0.31 points on the 80-point CAQ; 95%CI 0.001 to 0.62). More negative attitudes were associated with older age (− 1.55 points for each incremental decade from age 28; 95%CI − 2.67 to − 0.44), belief that adverse events are common with chiropractic care (− 1.41 points; 95% CI − 2.59 to − 0.23) and reported use of the research literature (− 6.04 points; 95% CI − 8.47 to − 3.61) or medical school (− 5.03 points; 95% CI  − 7.89 to − 2.18) as a source of knowledge on chiropractic. More positive attitudes were associated with endorsing a relationship with a specific chiropractor (5.24 points; 95% CI 2.85 to 7.64), family and friends (4.06 points; 95% CI 1.53 to 6.60), or personal treatment experience (4.63 points; 95% CI 2.14 to 7.11) as sources of information regarding chiropractic. (Table 4) The variance inflation factor was less than 2 for each independent variable, suggesting no issues with multicollinearity. Our model explained approximately 26% of the variation (adjusted *R*^2^ = 0.26) in family physician’s attitudes toward chiropractic.

**Table 4:** Variables associated with family physicians’ attitudes towards chiropractic (*n* = 379)

Variable	Unstandardized regression coefficient from univariable analysis (95% CI)	p-value	Unstandardized regression coefficient from multivariable analysis (95% CI)	p-value
Year of survey administration (2019 v. 2010)	0.16 (−0.17 to 0.48)	0.34	0.31 (0.001 to 0.62)	0.05
Age (for each 10-year increment from age 28)	−0.75 (−1.99 to 0.49)	0.24	−1.55 (−2.67 to − 0.44)	0.007
% of practice dedicated to musculoskeletal complaints	0.24 (−0.51 to 0.99)	0.53	0.16 (−0.51 to 0.83)	0.48
Belief that adverse events are common with chiropractic care	−1.42 (− 2.66 to − 0.19)	0.02	− 1.41 (− 2.59 to − 0.23)	0.02
Information source for chiropractic ^a^				
-Patient feedback	1.93 (− 1.24 to 5.10)	0.23	1.61 (− 1.29 to 4.51)	0.28
-Relationship with a specific chiropractor	7.74 (5.26 to 10.22)	< 0.001	5.24 (2.85 to 7.64)	< 0.001
-Research literature	−7.05 (−9.54 to −4.57)	< 0.001	−6.04 (−8.47 to −3.61)	< 0.001
-Personal treatment experience	8.65 (6.17 to 11.12)	< 0.001	4.63 (2.14 to 7.11)	< 0.001
-Family and friends	6.50 (3.85 to 9.14)	< 0.001	4.06 (1.53 to 6.60)	0.002
-Professors/supervisors/mentors	−5.12 (−8.41 to −1.83)	0.002	−2.23 (−5.37 to 0.92)	0.17
-Media	−4.17 (−7.57 to −0.77)	0.02	−1.39 (− 4.59 to 1.82)	0.40
-Medical school	−5.51 (−8.52 to −2.49)	< 0.001	− 5.03 (− 7.89 to − 2.18)	0.001

95% CI = 95% confidence interval

^a^ = each sub-category was entered individually into our generalized linear model as respondents could endorse multiple categories

## Discussion

Canadian family physician’s attitudes towards chiropractic have remained similar over the past decade. Most physicians held favourable perceptions of chiropractic, including the belief that chiropractic care is effective for some musculoskeletal complaints, provides a useful complement to conventional medicine, and can reduce family practitioner workload. However, attitudes are diverse, and respondents also highlighted several concerns, including uncertainty whether chiropractors provide evidence-base care, dependency on short-term symptom relief, and vaccine misinformation. The majority also agreed that practice diversity among chiropractors presented a barrier to interprofessional collaboration. Negative attitudes toward chiropractic care were associated with older age, belief that adverse events are common with chiropractic care, and reported use of the research literature or medical school as a source of knowledge on chiropractic. Endorsing a relationship with a specific chiropractor, family and friends, or personal treatment experience as sources of information were associated with more positive attitudes towards chiropractic.

### Strengths and limitations

Strengths of our study include random sampling of all Canadian family physicians, and survey design and administration consistent with best practices [27]. Our assessment of attitudes towards chiropractic was based on the CAQ, which has been validated among other groups of Canadian healthcare providers [28–30]. Our study does have limitations, including an overall response rate of 13%, which was lower for the re-administration of the survey. Non-responders may have differed systematically from responders, and the generalizability of our findings to family physicians practicing outside of Canada is uncertain. Our model explained 26% of the variation in respondent’s attitudes toward chiropractic, indicating that there remain additional variables of importance that our survey did not capture.

### Relevant literature

In August 2018, the Canadian Chiropractic Association (CCA) published a statement emphasizing a focus on promoting interprofessional collaboration [34], and the CCA advocates for integration of chiropractors into interprofessional health teams [35]. We found that although most Canadian family physicians endorse chiropractic care as a useful supplement to conventional medicine, only one in eight physicians reported working with a chiropractor, and practice diversity within the chiropractic profession was perceived as a barrier to interprofessional collaboration.

Most family physicians disagreed that chiropractic care was effective for non-musculoskeletal conditions, and systematic reviews on this topic have not found evidence to challenge this assertion [36–39]. Most respondents agreed that chiropractic care is effective for certain musculoskeletal complaints, and spinal manipulation, which is commonly provided by chiropractors, has received support for management of axial complaints from recent systematic reviews [40–45] and clinical practice guidelines [46–48]. Paradoxically, support from the scientific literature was a common reason given for referring patients for chiropractic care, while reliance on research literature for information on chiropractic was associated with more negative attitudes. Reasons for this disconnect are uncertain.

Close to half of respondents disagreed that chiropractic manipulation of the cervical spine was generally safe; however, although some observational studies have suggested a rare association with stroke [49–51], studies with greater methodologic safeguards against bias have failed to confirm either an association between greater utilization of chiropractic and risk of stroke [52], or an association between chiropractic care and an increased risk of stroke compared to care by primary care physicians [53, 54]. The associations reported in some studies between chiropractic care and stroke may be due to patients with early dissection-related symptoms seeking care prior to developing their strokes [55–57].

Musculoskeletal complaints, particularly low back pain, are common in primary care [58]. Our findings suggest that most Canadian family physicians believe chiropractors can provide effective care for some musculoskeletal complaints; however, many physicians are uncertain whether chiropractors treat in accordance with evidence-based practices and have concerns regarding the safety of cervical manipulation. The chiropractic profession may help address such concerns by continuing to assess the concordance between evidence and practice [59–62] and promoting greater standardization of care where important variability exists. Further research on the benefits and harms of cervical manipulation is needed to establish the appropriate role of this modality [63, 64].

Despite the many challenges that exist, there are good reasons to pursue improved relations between chiropractors and family physicians; interprofessional collaboration among healthcare providers is associated with improved patient satisfaction and outcomes [65, 66]. Moreover, preliminary evidence suggests that collaboration between chiropractors and physicians for shared patients may reduce use of prescription medication, including opioids, unnecessary imaging studies, and inappropriate referrals for surgical consultation [67, 68]. Efforts to improve relations may benefit from increased opportunities for family physicians and chiropractors to work together and learn from each other [7, 69, 70].

## Conclusions

Although generally positive, Canadian family physicians’ attitudes towards chiropractic range from very positive to extremely negative, and most physicians acknowledge that practice diversity within the chiropractic profession presents a barrier to interprofessional collaboration. Efforts to improve relations could include providing evidence-based information on chiropractic during medical training, and increased opportunities for family physicians and chiropractors to interact.

## Supplementary Information

**Additional file 1.**

## Data Availability

The blinded study data can be obtained from the corresponding author at: bussejw@mcmaster.ca
