# Supplementary material for: Attitudes towards chiropractic: a repeated cross-sectional survey of Canadian family physicians
Source: BMC Fam Pract. 2021 Sep 15;22:188. doi: 10.1186/s12875-021-01535-4 (PMC8442384; doi:10.1186/s12875-021-01535-4)
Supplement: Supplementary file 1 — Additional file 1. [file 12875_2021_1535_MOESM1_ESM.docx]

**Attitudes towards Chiropractic: A Repeated Cross-Sectional Survey of Canadian Family Physicians**

Jason W. Busse, DC, PhD;^1,2^ Sushmitha Pallapothu, BSc;^3^ Brian Vinh, BSc;^4^ Vivienne Lee, BSc;^5^ Lina Abril, BSc;^4^ Albana Canga;^6^ John J. Riva, DC, PhD;^7^ Daniel Viggiani, PhD;^8^ Marc Dilauro, MD;^9^ Marie-Pierre Harvey, DC, MSc;^10^ Isabelle Pagé, DC, PhD;^10,11^ Avneet Bhela, BSc;^3^ Serena Sandhu;^3^ Oluwatoni Makanjuola, BSc;^4^ Muhammad Taaha Hassan, BHSc;^4^ Ainsley Moore, MD;^7^ Claude A. Gauthier, DC;^12^ David J. Price, MD ^7^

1. Department of Health Research Methods, Evidence, and Impact, McMaster University, Hamilton, Ontario, Canada
2. Department of Anesthesia, McMaster University, Hamilton, ON, Canada
3. Department of Biology, McMaster University, Hamilton, ON, Canada
4. Department of Health Sciences, McMaster University, Hamilton, ON, Canada
5. The School of Interdisciplinary Science, McMaster University, Hamilton, ON, Canada
6. Institute for Work & Health, Toronto, Ontario
7. Department of Family Medicine, McMaster University, Hamilton, ON, Canada
8. Department of Kinesiology, University of Waterloo, Waterloo, ON, Canada
9. Michael G. DeGroote School of Medicine, McMaster University, Hamilton, ON, Canada
10. Département de chiropratique, Université du Québec à Trois-Rivières, Trois-Rivières, QC, Canada
11. Center for Interdisciplinary Research in Rehabilitation and Social Integration (Cirris), Quebec City, QC, Canada
12. Private practice, Gatineau, QC, Canada

**Funding:** The 2010 administration of our survey was supported by a $2,500 research grant from the Ontario Chiropractic Association. The funding source had no role in the conduct of the study; the collection, management, analysis, or interpretation of the data; or the preparation or approval of the manuscript.

**Corresponding Author:**

Jason W. Busse, Department of Anesthesia, Michael G. DeGroote School of Medicine, McMaster University, HSC-2V9, 1280 Main St. West, Hamilton, Canada, L8S 4K1

Tel: 905-525-9140 (x21731)

Fax: 905-523-1224

Email: bussejw@mcmaster.ca

**Keywords:** family medicine; chiropractic; survey; interprofessional relationship

Additional file 1: Family Physician’s Attitudes Towards Chiropractic Survey

Dear Dr. ________________________ ,

We are conducting a research study to gain a better understanding of Family Physician’s perceptions of chiropractic. Dr. Jason Busse is the primary investigator in this project. We are kindly asking for your cooperation in completing the attached **8-page survey**. Please answer each of the 50 questions listed in this survey. It will take you **less than 10 minutes** to respond by checking the **one box** (unless otherwise stated) that best reflects your answer to each question. The report of the results will be in an aggregate format without identifying information for particular participants. All responses will be kept confidential and the surveys themselves will be kept in a locked filing cabinet that is only accessible to the study investigators.

There may not be any direct benefit to you by participating in this survey. You can choose whether to complete this survey or not, and by returning the survey you indicate your consent to have your data included in this research study. If you volunteer to be in this study, you may withdraw your completed survey at any time without consequences of any kind by sending this request by e-mail (bussejw@mcmaster.ca). You may also refuse to answer any questions you don’t want to answer and still remain in the study.

- Please check this box if you **do not** wish to participate, and fax back this page to **xxx-xxx-xxx**, or send an e-mail indicating this choice (bussejw@mcmaster.ca), or you may also just discard the survey
- Please check this box if you **are not currently practicing family medicine**, and fax back this page to **xxx-xxx-xxx**, or send an e-mail indicating this choice (bussejw@mcmaster.ca), or you may also just discard the survey

We plan to send out this survey a total of 3 times to all intended participants, unless we receive a completed survey or an indication that a participant does not wish to participate. If you prefer, we can send the survey as an e-mail attachment and you can fill it out and return it by e-mail; please communicate this request by e-mail (bussejw@mcmaster.ca).

You are not waiving any legal claims, rights or remedies because of your participation in this research study. This study has been reviewed and received ethics clearance through the Hamilton Health Sciences/Faculty of Health Sciences Research Ethics Board.  If you have any questions regarding your rights as a research participant please contact the Office of the Chair at 905 521-2100, Ext. 42013.

- Please check this box if you wish to receive the results of this study

We greatly appreciate your time and cooperation. Once you have completed the survey, please fax back all pages to Jason Busse at: **xxx-xxx-xxx**.

Sincerely,

Jason Busse, DC, MSc, PhD

Associate Professor

Dept Anesthesia and Health Research Methods, Evidence and Impact

McMaster University

1. Gender:	□ Female	□ Male

2. Age: ­­_______

3. Country of Origin: □ Canada

□ USA

□ Other: _____________________________

4. Type of practice (**please check all that apply**):

□ Community

□ Hospital-based

□ Multidisciplinary

□ Private practice

□ Academic

□ Other: _______________________________________________

5. If you work in a multidisciplinary setting, is chiropractic care available?

- Yes
- No
- No, but I would be open to working with a chiropractor
- Unsure
- I do not work in a multidisciplinary setting

6. What best describes the proportion of your patient population with musculoskeletal complaints:

□ < 10%	□ 51% to 60%

□ 10% to 20%	□ 61% to 70%

□ 21% to 30%	□ 71% to 80%

□ 31% to 40%	□ 81% to 90%

□ 41% to 50%	□ >90%

7. What best describes your area of clinical interest: (**please check all that apply**)

□ General family medicine	□ Occupational medicine

□ Emergency medicine	□ Geriatrics

- Anesthesia	□ Sport medicine

□ Psychotherapy/Psychiatry	□ Pediatrics

□ Pain medicine	□ Palliative care

□ Obstetrics & gynecology

□ Other ____________________________________________________

8. Years in practice:	□ Less than 5 years

□ 5 to 10 years

□ 11 to 20 years

□ More than 20 years

9. How would you rate your knowledge of chiropractic?

□ I have no knowledge

□ A little knowledgeable

□ Moderately knowledgeable

□ Very knowledgeable

10. Have you ever received chiropractic care?

- Yes
- No

11. Did your medical training expose you to information about chiropractic?

- Yes, and the information was overall favorable
- Yes, and the information was overall neutral
- Yes, and the information was overall unfavorable
- No

12. Should medical training include exposure to information about chiropractic?

- Yes, definitely
- Possibly

□ Unsure

□ Probably not

□ No, definitely not

13. Do you feel comfortable discussing chiropractic care with your patients?

- Yes, definitely
- Somewhat

□ Unsure

□ Probably not

□ No, definitely not

14. Are you interested in learning more about chiropractic?

- Yes
- No
- Unsure

15. Do you have any training in spinal manipulation?

- Yes, and I use this modality with some of my patients
- Yes, but I do not use this modality with my patients
- No

16. Would you like to receive training in spinal manipulation?

- Yes
- No
- Unsure

17. How has your opinion of chiropractic been formed? (**Please check all that apply**)

□ Personal treatment experience	□ Patient feedback

□ Family and friends	□ Professors/supervisors/mentors

□ Research Literature	□ Relationship with chiropractor

□ Media	□ Residency

□ Medical school	□ Other _________________

□	I have no opinion on chiropractic

18. When were your opinions of chiropractic predominantly formed?

□ Before medical school

□ During medical school

□ After medical school

19. Should chiropractic care be available in a hospital setting?

- Yes, by primary access (no referral required)
- Yes, by physician referral only
- No
- Unsure

20. Should chiropractic care be available in multidisciplinary settings?

- Yes
- No
- Unsure

21. Should chiropractic care be covered, at least partially, by public/government funding?

- Yes
- No
- Unsure

22. How frequently do you refer your patients for chiropractic care?

□ Daily

□ Weekly

□ Monthly

□ Every year

□ Never

23. How many patients do you refer for chiropractic care in a typical year?

□ None

□ 1 to 10

□ 11 to 25

□ 26 to 50

□ More than 50

24. If you do refer patients for chiropractic care, what drives your referrals?

(**Please check all that apply**)

□ Patient request

- Non-response to medical treatment
- Literature supports chiropractic care for certain conditions
- Relationship with specific chiropractor
- My own positive experience as a chiropractic patient
- I do not refer patients for chiropractic care
- Other_____________________________________________________

25. Does practice diversity within the chiropractic profession present a barrier to greater collaboration with family physicians?

- Yes
- No
- Unsure

26. Do you perceive chiropractors as primary care providers?

- Yes, definitely
- Somewhat

□ Unsure

□ Probably not

□ No, definitely not

27. Would you like to receive a consultation note from a chiropractor who has seen one of your patients?

- Yes
- No
- Unsure
- Not applicable in my practice setting

28. Have patients been referred to your practice by chiropractors in order for you to refer them for imaging studies?

- Yes
- No
- Not applicable in my practice setting

29. Do you perceive that adverse events associated with chiropractic care are common?

- Yes
- Yes, but serious adverse events are rare
- No
- Unsure

30. Are you aware that chiropractors require a minimum of two years of undergraduate university level education prior to a four-year chiropractic education?

- Yes
- No

31. Chiropractors promote unnecessary treatment plans:

Strongly Agree □	Agree □	Undecided □	Disagree □	Strongly Disagree □

32. Chiropractors provide effective therapy for some musculoskeletal conditions:

Strongly Agree □	Agree □	Undecided □	Disagree □	Strongly Disagree □

33. Chiropractors make excessive use of radiographic imaging:

Strongly Agree □	Agree □	Undecided □	Disagree □	Strongly Disagree □

34. Chiropractors provide a patient centered approach:

Strongly Agree □	Agree □	Undecided □	Disagree □	Strongly Disagree □

35. When I see patients who have attended a chiropractor, I often have to spend time correcting erroneous information that they have received:

Strongly Agree □	Agree □	Undecided □	Disagree □	Strongly Disagree □

36. Chiropractic manipulation of the neck is generally a safe therapy for patients:

Strongly Agree □	Agree □	Undecided □	Disagree □	Strongly Disagree □

37. Chiropractors can provide effective therapy for some non-musculoskeletal conditions (e.g. asthma, infantile colic):

Strongly Agree □	Agree □	Undecided □	Disagree □	Strongly Disagree □

38. Family physicians may risk professional liability if they refer a patient to a chiropractor:

Strongly Agree □	Agree □	Undecided □	Disagree □	Strongly Disagree □

39. Chiropractors can reduce patient overload for family physicians for patients with musculoskeletal complaints:

Strongly Agree □	Agree □	Undecided □	Disagree □	Strongly Disagree □

40. Chiropractors provide patients with misinformation regarding vaccination:

Strongly Agree □	Agree □	Undecided □	Disagree □	Strongly Disagree □

41. Chiropractic provides effective therapy for post-surgical rehabilitation:

Strongly Agree □	Agree □	Undecided □	Disagree □	Strongly Disagree □

42. Chiropractors lack sufficient clinical training:

Strongly Agree □	Agree □	Undecided □	Disagree □	Strongly Disagree □

43. Chiropractic care is a useful supplement to conventional medicine:

Strongly Agree □	Agree □	Undecided □	Disagree □	Strongly Disagree □

44. Chiropractors engage in overly aggressive marketing:

Strongly Agree □	Agree □	Undecided □	Disagree □	Strongly Disagree □

45. Chiropractic includes ideas and methods from which conventional medicine could benefit:

Strongly Agree □	Agree □	Undecided □	Disagree □	Strongly Disagree □

46. The results of chiropractic manipulation are due to the placebo effect:

Strongly Agree □	Agree □	Undecided □	Disagree □	Strongly Disagree □

47. Chiropractors treat in accordance with evidence-based practice:

Strongly Agree □	Agree □	Undecided □	Disagree □	Strongly Disagree □

48. Chiropractic has no role in the routine care of my patients:

Strongly Agree □	Agree □	Undecided □	Disagree □	Strongly Disagree □

49. Chiropractic breeds dependency in patients on short-term symptomatic relief:

Strongly Agree □	Agree □	Undecided □	Disagree □	Strongly Disagree □

50. Overall, my impression of chiropractic is:

Very Good □	Good □	Undecided □	Poor □	Very Poor □

Please share with us any other thoughts you may have on chiropractic.

**- Thank You for Your Time -**

Additional file 1: Figure 1: 2010 Survey Administration Results

1000 Surveys sent by fax

238 No response after 3 waves of administration

634 Responses

196 Indicated they were not interested in completing the survey

187 Were ineligible to complete the survey:

121 no longer practicing family medicine

61 no longer at the contact office

4 retired from practice

1 on maternity leave

128 Fax number not in service

251 Completed surveys

Additional file 1: Figure 2: 2019 Survey Administration Results

2996 Surveys sent by fax

209 Fax number not in service

1991 No response after 3 waves of administration

796 Responses

276 Indicated that they were not interested in completing the survey

358 Were ineligible to complete the survey: not currently practicing, retired, maternity leave, no longer at the contact office

162 Completed Surveys

Additional file 1: Table 1: Family physician’s knowledge of and experience with chiropractic

| Year of administration | 2010 | 2019 |
| --- | --- | --- |
| No. of respondents | 251 | 162 |
| If you work in a multidisciplinary setting, is chiropractic care available, n (%)  yes  no  no, but I am open to working with a chiropractor  I do not work in a multidisciplinary setting | 35 (14%)  112 (45%)  14 (6%)  90 (36%) | 18 (11%)  81 (50%)  12 (7%)  51 (32%) |
| Have you received chiropractic care  yes  no | 94 (37%)  157 (63%) | 71 (44%)  91 (56%) |
| Are you interested in learning more about chiropractic, n (%)  yes  no  unsure | 88 (35%)  113 (45%)  50 (20%) | 63 (39%)  70 (43%)  29 (18%) |
| Did your medical training expose you to information on chiropractic, n (%)  yes, and the information was favorable  yes, and the information was neutral  yes, and the information was unfavorable  no | 9 (4%)  39 (16%)  33 (13%)  170 (68%) | 6 (4%)  30 (19%)  31 (19%)  95 (59%) |
| Should medical training include information on chiropractic, n (%)  definitely yes  possibly  unsure  probably not  definitely not | 134 (53%)  70 (28%)  27 (11%)  15 (6%)  4 (2%) | 80 (49%)  45 (28%)  18 (11%)  15 (9%)  4 (3%) |
| When were your opinions on chiropractic formed, n (%)  before medical school  during medical school  after medical school | 19 (8%)  21 (8%)  211 (84%) | 17 (10%)  19 (12%)  126 (78%) |
| How would you describe your knowledge of chiropractic, n (%)  I have no knowledge  a little knowledgeable  moderately knowledgeable  very knowledgeable | 12 (5%)  130 (52%)  94 (38%)  14 (6%) | 10 (6%)  79 (49%)  59 (36%)  14 (9%) |
| Are adverse events associated with chiropractic care common, n (%)  yes  yes, but serious adverse events are rare  no  unsure | 19 (8%)  72 (29%)  130 (52%)  30 (12%) | 15 (9%)  76 (47%)  14 (9%)  57 (35%) |
| Are you aware that chiropractors require a minimum of 2 years of undergraduate university level education prior to a 4-year chiropractic education, n (%)  yes  no | 66 (26%)  185 (74%) | 44 (27%)  118 (73%) |
| Do you feel comfortable discussing chiropractic care with patients, n (%)  definitely yes  somewhat  unsure  probably not  definitely not | 114 (46%)  81 (32%)  16 (6%)  29 (12%)  11 (4%) | 60 (37%)  67 (41%)  9 (6%)  14 (9%)  12 (7%) |
| Do you have training in spinal manipulative therapy, n (%)  yes, and I use this modality with some of my patients  yes, but I do not use this modality  no | 8 (3%)  16 (6%)  227 (90%) | 2 (1%)  6 (4%)  154 (95%) |
| Would you like to receive training in spinal manipulative therapy, n (%)  yes  no  unsure | 30 (12%)  182 (73%)  39 (15%) | 21 (13%)  122 (75%)  19 (12%) |

Additional file 1: Table 2: Family physician’s beliefs regarding integration of chiropractic care

| Year of administration | 2010 | 2019 |
| --- | --- | --- |
| No. of respondents | 251 | 162 |
| Should chiropractic care be available in multidisciplinary settings, n (%)  yes  no  unsure | 35 (14%)  140 (56%)  76 (30%) | 27 (17%)  87 (54%)  45 (28%) |
| Should chiropractic care be available in a hospital setting, n (%)  yes, by primary access  yes, by physician referral  no  unsure | 14 (6%)  50 (20%)  87 (35%)  100 (40%) | 20 (12%)  19 (12%)  65 (40%)  58 (36%) |
| Should chiropractic care be covered, at least partially, by government funds, n (%)  yes  no  unsure | 83 (33%)  88 (35%)  80 (32%) | 61 (38%)  49 (30%)  52 (32%) |
| Does diversity within chiropractic present a barrier to collaboration, n (%)  yes  no  unsure | 160 (64%)  31 (12%)  60 (24%) | 85 (52%)  30 (19%)  47 (29%) |
| Do you perceive chiropractors as primary care providers, n (%)  yes, definitely  somewhat  unsure  probably not  definitely not | 42 (17%)  66 (26%)  20 (8%)  52 (21%)  71 (28%) | 26 (16%)  43 (27%)  12 (7%)  30 (19%)  51 (31%) |
| Would you like a consultation note from a chiropractor if they see your patient, n (%)  yes  no  unsure  not applicable to my practice setting | 195 (78%)  33 (13%)  19 (8%)  4 (2%) | 140 (86%)  13 (8%)  8 (5%)  1 (1%) |
| Have chiropractors referred you patients to send for imaging studies, n (%)  yes  no  not applicable to my practice setting | 202 (81%)  45 (18%)  4 (2%) | 122 (75%)  36 (22%)  4 (3%) |
